# Type 2 Diabetes and Cardiovascular Risk in Women 2016

**DOI:** 10.1155/2017/6905697

**Published:** 2017-07-04

**Authors:** Alexandra Kautzky-Willer, Giovannella Baggio, Maria Chiara Rossi, Annunziata Lapolla, Giuseppina T. Russo

**Affiliations:** ^1^Gender Medicine Unit, Division of Endocrinology and Metabolism, Department of Internal Medicine III, Medical University of Vienna, Vienna, Austria; ^2^Gender Medicine Institute, Gars am Kamp, Austria; ^3^Chair of Gender Medicine, Molecular Medicine Department, University of Padova, Padova, Italy; ^4^Center for Outcomes Research and Clinical Epidemiology (CORESEARCH), Pescara, Italy; ^5^Department of Medicine, University of Padova, Padova, Italy; ^6^Department of Clinical and Experimental Medicine, University of Messina, Messina, Italy

Due to population growth and ageing, diabetes is now among the 8 leading causes of death [1]. Thus, type 2 diabetes comprising the majority of diabetic patients is one of the most important NCDs and its steep rise and associated complications go along with mounting evidence of clinically important sex and gender differences [2]. Genetic background, lifestyle, epigenetics, and environment contribute to the pandemic increase with important biological and psychosocial risk factors of men and women. Overall, globally, more men are diagnosed with diabetes as there were 15.7 million more men than women with diabetes in 2015 [3]. There are large sex-ratio differences regarding diabetes across countries which parallel those of obesity, the most prominent risk factor in both sexes. Type 2 diabetes is more frequently and at a younger age and lower body-mass-index (BMI) diagnosed in males as men usually feature more visceral fat and higher degree of insulin resistance compared to women of comparable age and BMI. However, waist is a better predictor of diabetes and cardiovascular disease in women who also have a greater relative risk of cardiovascular complications and mortality in the presence of prediabetes, the metabolic syndrome, or overt diabetes [4, 5]. Altogether, diabetic women bear a greater risk to suffer and die from myocardial infarction or stroke than men in comparison to same sex nondiabetic subjects [6].

Diversities in biology, culture, lifestyle, and socioeconomic status impact sex dimorphism in clinical presentation of type 2 diabetes. Biological differences comprise differences in body composition, glucose and fat metabolism, energy balance, and neuroendocrine regulation [2]. In particular, reproductive history and reproductive factors are important for evaluation of diabetes and cardiovascular risk. Thus, women with early menarche, irregular cycles, or the PCOS were shown to be at higher risk. However, the most important risk factor in women appears to be gestational diabetes which affects approximately 10% of all pregnant women and is associated with both acute and long-term complications in mothers and offspring. Therefore, sex-specific guidelines for stroke prevention in women were recently released outlining the importance of gestational diabetes and preeclampsia as sex-specific risk factors and the impact of diabetes, depression, and psychosocial stress as risk factors particularly in females [7]. Differences in therapy and interventions further contribute to different outcomes in diabetic patients with greater disparities in women [8].

Therefore, the issue of prevention and therapy of cardiovascular disease is of utmost importance for health-related quality of life of diabetic women. To this end, this special series will cover interesting papers on this important topic summarizing current evidence and further expanding our present knowledge.

One paper describes the incidence of stroke and stroke subtypes derived from the stroke and diabetes surveillance system in China. L. Guo et al. report almost fourfold excess risk in diabetic patients, especially in females and particularly regarding the subtype cerebral infarction.

Another study by M. Leutner et al. analysed metabolic and vascular characteristics of treated hyperlipidemic men and women. Overall, vascular morphology, insulin sensitivity, and glucose tolerance did not differ between sexes although women had a more favourable lipid profile and better liver enzymes.

In addition, a review by G. T. Russo et al. will address the important topic of osteoporosis and fracture risk based on experimental and clinical evidence in men and women with diabetes. Both sex differences in pathophysiology and lifestyle and gender implications including the side effects of glucose-lowering drug therapies will be discussed.

Further, a review by S. Burlina et al. will present and discuss the current evidence of cardiovascular risk in women with gestational diabetes. This is important as this growing number of women could present an ideal group for sex-specific diabetes and cardiovascular prevention programs. Early identification of those women at the highest risk could reduce the burden of transgenerational diabetes.

We hope that this special series will further highlight the importance of cardiovascular risk in diabetic women, stimulate new research, and contribute to better awareness and care.

*Alexandra Kautzky-Willer* *Alexandra Kautzky-Willer* *Giovannella Baggio* *Giovannella Baggio* *Maria Chiara Rossi* *Maria Chiara Rossi* *Annunziata Lapolla* *Annunziata Lapolla* *Giuseppina T. Russo* *Giuseppina T. Russo*
